# Supplementary material for: The Differences of Nutrient Components in Edible and Feeding Coix Seed at Different Developmental Stages Based on a Combined Analysis of Metabolomics
Source: Molecules. 2023 Apr 27;28(9):3759. doi: 10.3390/molecules28093759 (PMC10180337; doi:10.3390/molecules28093759)
Supplement: Supplementary file 1 [file molecules-28-03759-s001.zip › molecules-2250878-supplementary/Supplementary materials/Supplement.pdf]

The differences of nutrient components in edible and feeding Coix seed at different developmental stages based of combined analysis of metabolomics

Xiaoyan Wei [1,2], Yong Li [2], Shufeng Zhou [3], Chao Guo [3], Xiaolong Dong [3], Qishuang Li [2], Juan Guo [2], Yanan Wang [2,*], and Luqi Huang [1,2,*]

[1] College of Chinese Medicinal Materials, Jilin Agricultural University, Changchun 130118, China

[2] State Key Laboratory Breeding Base of Dao-di Herbs, National Resource Center for Chinese Materia Medica, China Academy of Chinese Medical Sciences, Beijing 100700, China

[3] State Key Laboratory of Exploration and Utilization of Crop Gene Resources in Southwest China, Key Laboratory of Biology and Genetic Improvement of Maize in Southwest Region, Ministry of Agriculture, Maize Research Institute of Sichuan Agricultural University, Chengdu 611130, China

Supplementary materials:

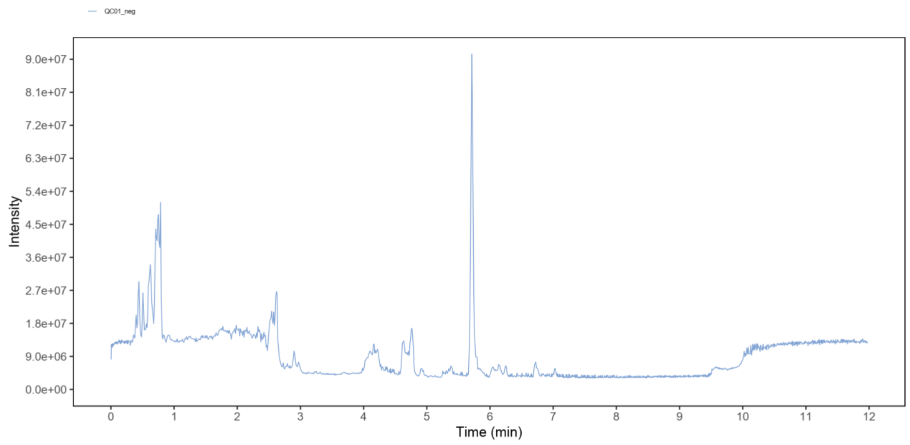

Figure S1. TIC diagram of negative ion mode detected by UHPLC-QTOF-MS of the quality control (QC) sample.

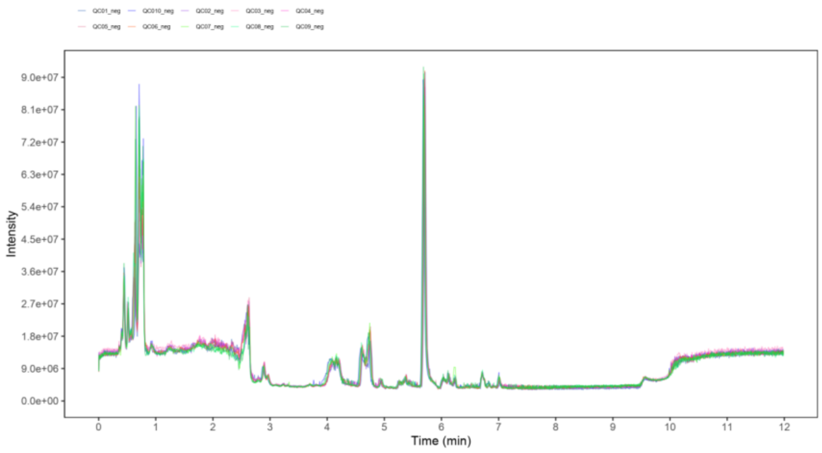

Figure S2. TIC diagram of all QC samples in negative ion mode.

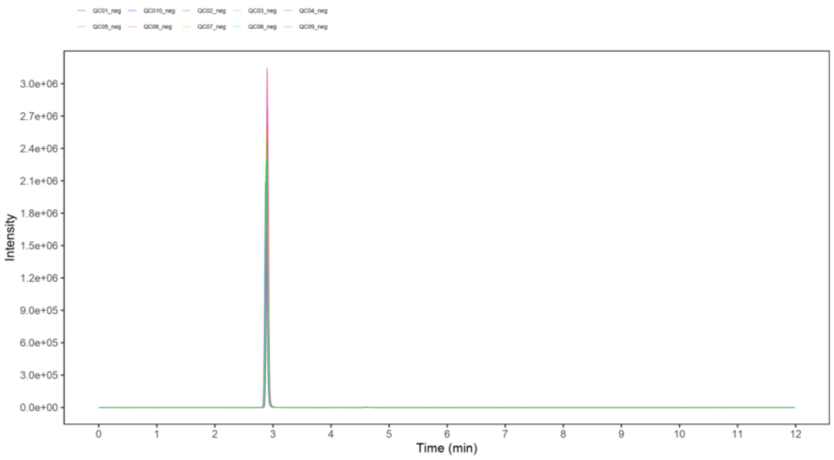

Figure S3. EIC diagram of internal standard negative ions in QC sample.

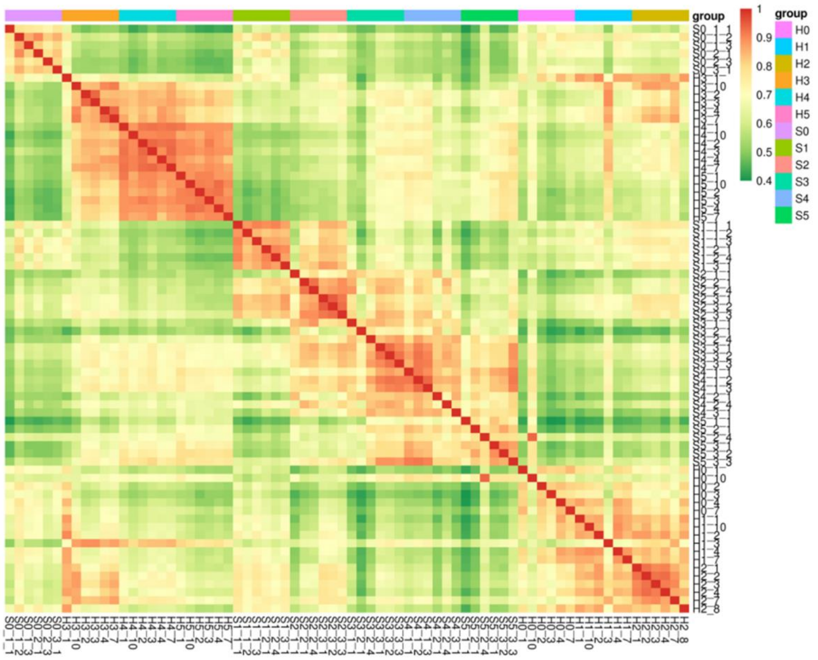

Figure S4. Correlation analysis of all *Coix lachryma-jobi* L. samples.

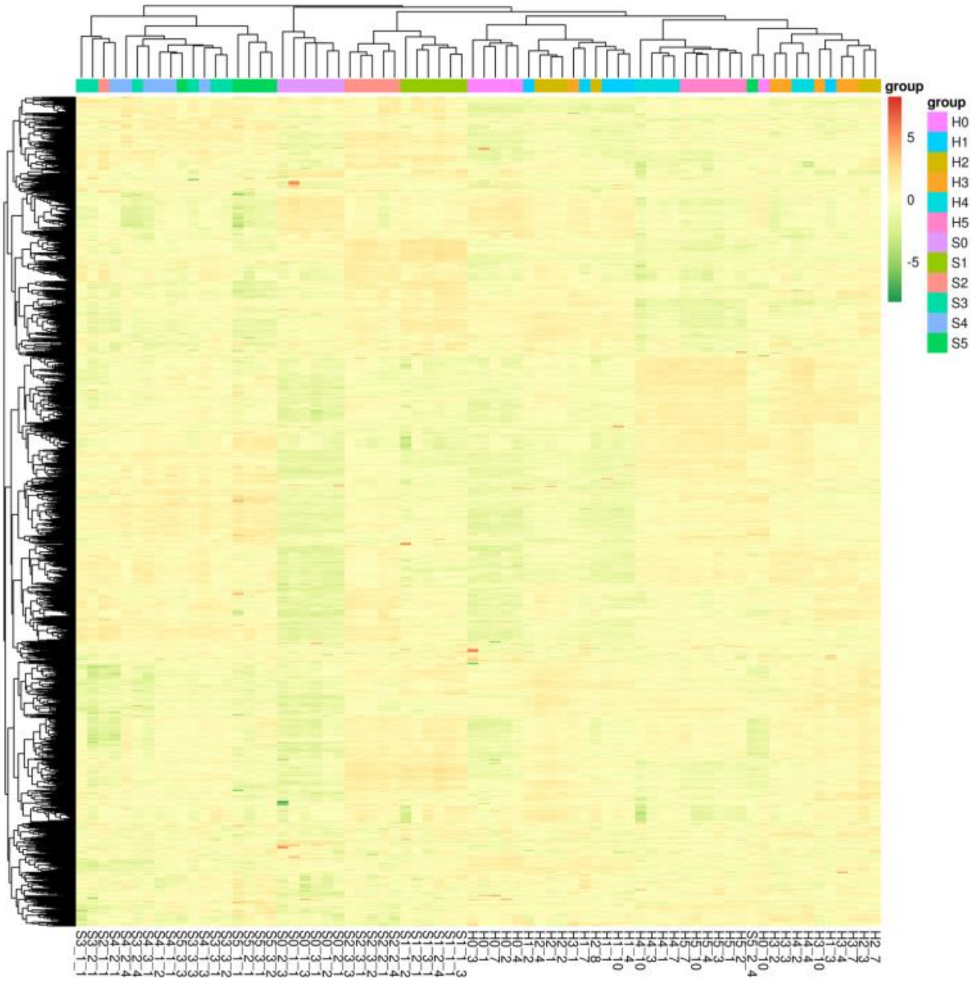

Figure S5. Heatmap of all Coix seeds at different developmental stages.
